# A Re-audit of the Management of Diabetic Ketoacidosis (DKA): The Importance of the Seven-Day Working Inpatient Diabetes Specialist Nurse Service

**DOI:** 10.7759/cureus.56723

**Published:** 2024-03-22

**Authors:** Roobala K Periyasami, Andrew Pei Wern Lim, Dineth C Jayatilake, Samson O Oyibo

**Affiliations:** 1 Acute Internal Medicine, Peterborough City Hospital, Peterborough, GBR; 2 General Internal Medicine, Peterborough City Hospital, Peterborough, GBR; 3 General Medicine, Peterborough City Hospital, Peterborough, GBR; 4 Diabetes and Endocrinology, Peterborough City Hospital, Peterborough, GBR

**Keywords:** treatment guidelines, hypoglycemia, transient hypokalemia, 7-day working, inpatient diabetes specialist nurse, re-audit, diabetic ketoacidosis (dka)

## Abstract

Background

Diabetic ketoacidosis (DKA) is a life-threatening metabolic emergency due to insulin deficiency in patients with diabetes mellitus. The United Kingdom national survey and local audits of the management of DKA have revealed several areas of suboptimal care, and room for improvement, necessitating the need for intensified education, updating local guidelines, and increased recruitment of seven-day working inpatient diabetes specialist nurses. Therefore, this project aimed to re-audit our adherence to the DKA treatment guidelines.

Methodology

A retrospective re-audit examining patient admissions with DKA between October 2022 and September 2023. A list of 18 standards/criteria, adopted from the Joint British Diabetes Society (JBDS) DKA treatment guidelines was used for this re-audit. Results were compared with that of the previous audit.

Results

We had 126 patients admitted with DKA between October 2022 and September 2023. There were 62 males and 64 females with an average (range) age of 46.5 (19-92) years. Eighty percent had type 1 diabetes, and common precipitating factors for admission included infection and poor adherence to insulin treatment. The median (IQR) length of hospital stay was 2.1 (1.0-5.1) days. Compared to the previous audit, improvements occurred in 11 of 18 standards/criteria. This included timely commencement of intravenous fluids and fixed-rate insulin, commencing glucose infusion to prevent hypoglycemia, potassium replacement, continuation of long-acting insulin during treatment, timely conversion to variable-rate insulin infusion, and conversion to the usual subcutaneous insulin regimen. Additionally, 124 patients (98.4%) were reviewed at least once by the inpatient diabetes specialist nurses (DSN) during their admission. Complications of treatment, namely, iatrogenic hypoglycemia and transient hypokalemia occurred in 13 (10.3%) and 40 (31.7%) patient admissions, respectively.

Conclusions

This re-audit demonstrated improved adherence to the guidelines during several steps in the management of DKA. It also demonstrated room for improvement regarding other aspects of care. The importance of continued education, accurate documentation, and the presence of seven-day working inpatient DSN cover cannot be overemphasized.

## Introduction

Diabetic ketoacidosis (DKA) is a life-threatening metabolic emergency due to insulin deficiency in patients with diabetes mellitus requiring prompt treatment [1,2]. The DKA diagnostic criteria, which include a blood glucose concentration >11.0 mmol/L or known to have diabetes mellitus, capillary ketones >3 mmol/L or significant ketonuria (2+ or more) and a bicarbonate concentration <15.0 mmol/L and/or venous pH <7.3, and accompanying symptoms, such as vomiting, dehydration, hyperventilation and drowsiness, are taken into account in the diagnosis and initial management [1,2]. The Joint British Diabetes Society has set out clear guidelines for the management of DKA [2]. The management of diabetic ketoacidosis is time-consuming and labor-intensive, but strict adherence to protocol is crucial for reducing morbidity, mortality, and hospital length of stay [1,2]. Patients should have intravenous access, be given fluids and insulin, and have routine investigations performed within an hour of assessment [2].

The national survey of the management of DKA carried out in the United Kingdom in 2014 revealed that several areas of management were suboptimal despite the widespread adoption of national guidance [3]. Our previous local audit of the management of diabetic ketoacidosis also demonstrated room for improvement. Since then, education has intensified and local guidelines were updated in line with revised national guidance and there was increased recruitment of seven-day-working inpatient diabetes specialist nurses (DSN) who provided continuous education, monitoring, and ensuring adherence to the treatment guidelines during acute admissions.

We aimed to re-assess our adherence to the DKA treatment guidelines by performing a re-audit and comparing results with that of the previous audit. We wanted to assess for improvements in our management of DKA and assess if there were any complications of treatment such as hypoglycemia or hypokalemia.

## Materials and methods

Patients who were admitted with diabetic ketoacidosis (DKA) between the months of October 2022 and September 2023 were included in this retrospective re-audit. The list of patients who met this inclusion criteria was obtained from our Patient Information Services. Patients below the age of 16 years and pregnant females were not included in this audit cycle. Clinical information regarding the DKA admission was obtained from the patient records and inputted into a Microsoft Excel spreadsheet (Microsoft Corporation, Redmond, WA, USA). The list of standards/criteria employed for this re-audit and previous audit was adopted from the Joint British Diabetes Society (JBDS) DKA treatment guidelines [2]. There were 18 standards/criteria. Each patient admission had a “yes”, “no” or “not applicable” answer to whether a standard/criterion was met. Data collection and collation were carried out by four auditors over three months. This retrospective re-audit was approved by and registered with our Quality Governance and Compliance Department (Project Code: 4093).

Data analysis

The proportion of patient admissions that met a standard/criterion was calculated by dividing the number of patients who had a ”yes” answer to that standard/criterion being met by the total number of applicable patient admissions. The adherence score (percentage) was calculated by multiplying the derived proportion by 100. The data were presented as whole numbers and percentages. Using our in-house guide, based on national guidance, a score of less than 75% was considered not adherent, 75-90% was partially adherent, and above 90% was adherent, with 100% being fully adherent [4]. The target score for each standard/criterion was set a priori to 90-100%. An increase in the adherence score (positive difference) over the first audit was taken as an improvement while a negative difference indicated challenges. The chi-squared test for differences in proportions was used to assess whether the observed differences were statistically significant [5,6]. A p-value of less than 0.05 was considered statistically significant.

## Results

One hundred and twenty-six patients were admitted because of DKA between October 2022 and September 2023. The cohort had an average (range) age of 46.5 (19-92) years, and the median (inter-quartile range) length of hospital stay was 2.1 (1.0-5.1) days. Table 1 shows further baseline characteristics for the 126 patient admissions.

**Table 1:** Baseline data for the 126 patient admissions n (%): number (percentage); DKA: diabetic ketoacidosis

Characteristics	n (%)
Males	62 (49.2)
Females	64 (50.8)
Type 1 diabetes	101 (80.2)
Type 2 diabetes	25 (19.8)
Precipitating cause found for DKA	126 (100)
- Localized infection	43 (34.1)
- Poor adherence to insulin treatment	33 (26.2)
- New diagnosis of type 1 diabetes	8 (6.3)
- Sepsis	7 (5.6)
- Alcohol	3 (2.4)
- Other illnesses, such as gastrointestinal bleeding illnesses, dehydration, poor oral intake, etc.	32 (25.4)

Compared to the previous audit, there were notable increases in the percentage of patient admissions that met 11 out of the 18 standards/criteria. This included documenting the diagnosis of DKA and commencing intravenous fluid and intravenous fixed-rate insulin within an hour of assessment, potassium replacement according to guidelines, the use of 10% glucose infusion to prevent iatrogenic hypoglycemia, the continuation of long-acting insulin during DKA treatment, the conversion from fixed-rate insulin to variable-rate insulin infusion upon resolution of the DKA, conversion to usual subcutaneous insulin regimen once the patient was eating and drinking, patients being reviewed by the inpatient diabetes specialist nurses (DSN) within 24 hours of assessment. One hundred and twenty-four patients (98.4%) had at least one documented review by the inpatient DSN during their admission. Overall, a partial adherence to adherence status was given to 14 out of the 18 standards/criteria. Only the percentage increases for commencing fixed-rate insulin within the hour, potassium replacement according to guidelines, and prompt conversion to the usual subcutaneous insulin regimen were statistically significant. The standards/criteria used for this re-audit and the percentage number of patients who had each standard/criterion met are displayed in Table 2. Results of the previous audit are included for comparison.

**Table 2:** Results and comparison between the re-audit and the previous audit N: total number of patient-admission applicable, n (%): number of patient admissions that met the standard/criterion (adherence score in percentage), DSN: diabetes specialist nurses, DKA: diabetic ketoacidosis *The inpatient DSN reviewed 98.4% of patients at least once during their admission.

Audit standards/criteria	1^st^ audit		Re-audit		Difference in adherence (%)	P-value
	N	n (%)	N	n (%)		
Diagnosis according to DKA criteria?	148	121 (82)	126	112 (89)	+7	0.1
Fluids started within the hour of diagnosis?	148	120 (81)	126	109 (87)	+6	0.18
Fixed-rate insulin within the hour of diagnosis?	148	103 (70)	126	105 (84)	+14	0.007
IV fluids according to Trust guidelines?	148	118 (80)	121	88 (73)	-7	0.17
Fixed-rate insulin according to Trust guidelines?	148	120 (81)	121	84 (70)	-11	0.035
Potassium replacement according to guidelines?	148	98 (66)	126	97 (77)	+11	0.046
Capillary blood glucose done hourly?	148	120 (81)	126	94 (75)	-6	0.23
Was urine output documented?	148	123 (83)	126	73 (58)	-25	<0.0001
Was 10% glucose started when capillary blood glucose < 14 mmol/l?	148	107 (72)	126	94 (75)	+3	0.58
Was the patient’s long-acting insulin continued?	128	105 (82)	115	103 (90)	+8	0.075
Resolution of DKA confirmed in the notes?	148	117 (79)	126	94 (75)	-4	0.43
VRIII started on the resolution of DKA?	148	114 (77)	126	106 (84)	+7	0.15
Promptly converted to usual subcutaneous insulin when eating/drinking?	140	124 (89)	121	118 (98)	+9	0.004
Senior review within 12 hours?	148	135 (91)	126	116 (92)	+1	0.77
Inpatient DSN review within 24 hours?*	148	126 (85)	126	115 (91)	+6	0.13
Referred to inpatient DSN?	145	123 (85)	126	116 (92)	+7	0.075
Pre-discharge review by inpatient DSN?*	139	115 (83)	116	95 (82)	-1	0.83
Accurate discharge letter (insulin, dose, care plan, follow-up, etc.)?	148	118 (80)	126	73 (58)	-22	0.0001

When compared to the previous audit, there was a decline in the percentage of patient admissions meeting the standards/criteria surrounding, continuation of intravenous fluids and fixed-rate insulin infusions according to guidelines, capillary blood glucose assessment hourly, documentation of urine output and confirmation of resolution of DKA in the notes. The accuracy of the patient discharge letters was also compromised. However, only the percentage reductions in continuing fixed-rate insulin according to guidelines, documenting urine output, and accuracy of discharge letters were statistically significant.

There were also complications of DKA treatment, namely, hypoglycemia and transient hypokalemia. The median (interquartile range) potassium level during all episodes of transient hypokalemia among the cohort was 3.2 (3.0-3.3) mmol/L. Table 3 shows data concerning complications of DKA treatment and mortality data. There were no deaths due to the treatment of DKA.

**Table 3:** Complications of DKA treatment and mortality data n (%): number (percentage); DKA: diabetic ketoacidosis *Deaths were due to other comorbidities, such as metastatic cancer, pneumonia, and sepsis, in patients after the resolution of DKA.

Complications of DKA treatment	Frequency (n (%))
- Iatrogenic hypoglycemia	13 (10.3)
- Transient hypokalemia	40 (31.7)
Mortality data	Frequency (n (%))
- Deaths*	7 (5.6)

## Discussion

This project aimed to re-assess our adherence to the DKA treatment guidelines after continued education and increasing the presence of seven-day working inpatient DSN cover: assessing for improvements compared to the previous audit and assessing for complications of treatment such as hypoglycemia or hypokalemia.

Improvements

This re-audit has demonstrated improvement in terms of adherence scores for several important steps in the management of DKA when compared to the previous audit. A partially adherent to adherent status was assigned to 14 of the 18 standards/criteria assessed, with five having an adherence score of 90% or above as opposed to only one standard having this score in the first audit.

Prompt commencement of intravenous fluid and insulin, appropriate potassium replacement, and timely use of 10% glucose infusion to prevent iatrogenic hypoglycemia, continuation of long-acting insulin during DKA treatment, timely conversion to variable-rate insulin infusion, timely conversion to patient’s usual subcutaneous insulin regimen, and inpatient diabetes nurse review all had improvements in the adherence scores compared to the first audit. The adherence score for patients having a senior review within 12 hours remained the same (above 90%). We believe continued education and the seven-day presence of the inpatient DSN contributed to the observed improvements in adherence scores.

Although not statistically significant, there was an increase in the adherence score for patients being seen by the inpatient DSN within 24 hours and a reduction in the adherence score for patients having a pre-discharge DSN review. The inpatient DSN was able to see 115 (91%) patients within 24 hours of initial assessment and was able to see 95 (82%) patients for a pre-discharge review. The observed reduction in the adherence score for patients having a pre-discharge DSN review could be explained by a significant number of patients being discharged within two days providing only a small window for them to have both an early review and a pre-discharge review. However, 124 (98.4%) patients were reviewed at least once by the inpatient DSN during their admission.

Our inpatient DSN works seven days a week (0800-1600 hours Monday to Friday and 0700-1500 hours on Saturday and Sunday). In addition to ward duties, they ensure early and rapid assessment and encourage adherence to guidelines during the treatment of DKA in the emergency department. They assist in monitoring parameters and complications of treatment, ensuring the maintenance of basal insulin to prevent reoccurrence of DKA, escalating difficult cases to the diabetes consultant, new insulin commencement, and safe discharge of patients after treatment. They provide education and sick-day rules to the patient, ensure adequate insulin and devices on discharge, and facilitate transfer/hand-over to the community diabetes team and outpatient clinics [7,8].

Challenges

This re-audit demonstrated room for improvement to reach a level above 90% or full adherence in several aspects, but more so for areas where we were non-adherent to partially adherent. These areas included continuation of intravenous fluids and fixed-rate insulin infusions according to guidelines, monitoring hourly capillary blood glucose levels, when documenting urine output, and writing accurate patient discharge letters.

Fluid regimens may have been altered in special circumstances, such as in elderly frail patients, young adults, patients with heart failure or kidney failure, and patients with other serious comorbidities [2]. Additionally, regular documentation may not have been synchronous with actual practice. Hence, the importance of accurate and timely documentation during practice and when deviating from protocol for clinical reasons cannot be overemphasized.

Fixed-rate insulin infusion, once started, should be continued until the resolution of DKA before conversion to variable-rate insulin [2]. This audit demonstrated an improvement in the adherence score for starting the fixed-rate insulin within the hour but a reduction in the score for continuing the fixed-rate insulin infusion according to guidelines. On closer inspection, some of these cases had mild DKA and were rapidly or directly converted to variable-rate insulin infusion. Poor documentation was another reason contributing to the reduction in the adherence score. This again emphasizes the importance of timely documentation and more so, when deviating from protocol for clinical reasons. This will be examined in more detail during the next audit.

Most patients did not have urinary catheterization during the treatment of DKA. This is only required if a patient is incontinent or anuric [2]. However, monitoring urine output is very important in detecting acute kidney injury, monitoring for fluid overload, and during potassium replacement. Therefore, work is required to improve the documentation of urine output during the management of DKA.

Several discharge letters did not mention the diagnosis of DKA, whether the patient was aware of changes to their insulin regimen, the correct insulin delivery device, or a clear follow-up plan. Work is required to improve the accuracy and quality of discharge letters for patients after admission with DKA.

Complications of DKA treatment

Hypoglycemia occurred during treatment in 13 (10.3%) patient-admissions. The occurrence of hypoglycemia during the treatment of DKA could be due to a combination of mistimed conversion from fixed-rate insulin to variable-rate insulin infusion, mistimed addition of 10% glucose infusion to run concurrently once capillary glucose falls below 14 mmol/L, and irregular capillary blood glucose measurements. Documented evidence of transient hypokalemia occurring during treatment affected 40 (31.7%) patient admissions. Potassium results were obtained mainly from the blood gas analyzer and observed hypokalemic episodes were both mild and transient (i.e., one low level out of several normal results). Blood gas analyzers provide fast reliable bedside results during the management of DKA [2,9]. On the other hand, it was difficult to quantify iatrogenic hyperkalemia because several patients had hyperkalemia on admission (i.e., before treatment). Our incidence rate of both hypoglycemia and transient hypokalemia during treatment was less than that noted in the national survey report of 2014, where the incidence rate was 27.6% and 55%, respectively [3]. Further work is required to prevent iatrogenic hypoglycemia and transient hypokalemia during the management of DKA.

Mortality data

There were no reported deaths due to the treatment of DKA. Previous studies have demonstrated that mortality from a single DKA admission is less than 1% [10]. In a six-year study involving 628 DKA admissions, the initial inspection identified no deaths during inpatient admission for the management of DKA. However, on closer inspection, one death was discovered where DKA was reported as a contributory factor: giving an inpatient mortality rate of 0.16% [10]. Our study identified seven inpatient deaths that were not related to DKA management, but other underlying co-morbidities.

Similar studies

The results of this re-audit bear some similarities with those of other audits that have been published since the national survey was carried out in 2014. However, these audits involved smaller patient numbers, which make direct comparison difficult [11-13]. As previously mentioned, the management of DKA is time-consuming and labor-intensive. It is, therefore, not impossible to fall behind slightly with numerous infusions, hourly measurements, and hourly documentation in a busy emergency department, even with the use of bespoke bedside charts. This highlights the importance of having one-to-one nursing in a high-dependency unit and the availability of seven-day working inpatient DSN cover for the management of patients admitted with DKA. The prevention of complications of treatment and ensuring full adherence to the treatment guidelines to optimize clinical outcomes remain the goals during the management of DKA.

Study limitations

There are several limitations to this study. First, it was sometimes difficult to interpret the timing of observations, fluid, and insulin administrations on the charts mainly because of inadequate/unclear/illegible documentation. Electronic prescribing once started in our establishment would reduce such difficulties. Second, we did not have full information on all the “not-applicable” groups for each standard/criterion and the reason why they were deemed not applicable. Although these represented small numbers, the full reasons for being not applicable should be included in future re-audits. Third, the data was collected and collated by four auditors with varied data collecting/collating skills. Therefore, the quality of the data can vary according to who is collating the data. However, to reduce such variances, multiple team meetings were held to improve uniformity during the study period. Finally, findings from this study are representative of a single setting and limited period (1 year) and may not apply to other healthcare institutions. Further national surveys are required to assess these findings.

## Conclusions

We have presented our re-audit of the management of DKA in a busy district hospital and demonstrated improvement in adherence to the guidelines during several steps. We have highlighted room for improvement regarding the continuation of intravenous fluids and fixed-rate insulin infusions, regular blood glucose monitoring, accurate documentation, especially when deviating from the protocol for clinical reasons, and the provision of accurate discharge letters. We have also demonstrated that our complication rates of hypoglycemia and hypokalemia are less than those reported in the previous national survey.

The importance of continued education, accurate documentation, and the presence of seven-day working inpatient DSN cover in enhancing adherence to the DKA management guidelines cannot be overemphasized.

## References

[1] Savage MW, Dhatariya KK, Kilvert A (2011). Joint British Diabetes Societies guideline for the management of diabetic ketoacidosis. Diabet Med.

[2] (2024). Joint British Diabetes Societies Inpatient Care Group. The management of diabetic ketoacidosis in adults. March.

[3] Dhatariya KK, Nunney I, Higgins K, Sampson MJ, Iceton G (2016). National survey of the management of diabetic ketoacidosis (DKA) in the UK in 2014. Diabet Med.

[4] (2024). Health Quality Improvement Partnership. Documenting local clinical audit: a guide to reporting and recording. April.

[5] Campbell I (2007). Chi-squared and Fisher-Irwin tests of two-by-two tables with small sample recommendations. Stat Med.

[6] Richardson JT (2011). The analysis of 2 × 2 contingency tables—yet again. Stat Med.

[7] McHoy A (2003). The role of the inpatient DSN. J Diabetes Nursing.

[8] Alabraba V, Floyd E, Wallymahmed M (2010). Delivering a diabetes inpatient specialist service: the Aintree experience. J Diabetes Nursing.

[9] Mahmoud H, Jaffar Z, Al Alawi YM (2022). Accuracy of potassium measurement using blood gas analyzer. Cureus.

[10] Gibb FW, Teoh WL, Graham J, Lockman KA (2016). Risk of death following admission to a UK hospital with diabetic ketoacidosis. Diabetologia.

[11] Mccarthy AM, Crowley R (2021). An audit on the management of diabetic ketoacidosis during the COVID-19 pandemic in St. Vincent’s University Hospital, Dublin, Ireland. Diabetes.

[12] Ssemmondo E, Wong HH, Moteea S, Abobaker A, Pawlak T (2021). A reaudit testing the adherence to the local trust protocol detected delay in potassium replacement during acute management of diabetic ketoacidosis. Int J Clin Pract.

[13] Kurdi H, Pinto LP, Smeeton FJ (2017). A re-audit of the management of diabetic ketoacidosis after the introduction of a local protocol based on the JBDS guidelines: then and now. Br J Diabetes.

